# Effects of Increasing Oral Deoxynivalenol Gavage on Growth Performance, Blood Biochemistry, Metabolism, Histology, and Microbiome in Rats

**DOI:** 10.3390/biology13100836

**Published:** 2024-10-18

**Authors:** Jin-Young Jeong, Junsik Kim, Minji Kim, Seong-Hoon Shim, Cheolju Park, Sungju Jung, Hyunjung Jung

**Affiliations:** 1 Animal Nutrition and Physiology Division, National Institute of Animal Science, Wanju 55365, Republic of Korea; kkk940326@korea.kr (J.K.); mjkim00@korea.kr (M.K.); kanji0616@korea.kr (S.-H.S.); hyjjung@korea.kr (H.J.); 2 Division of Animal Science, College of Agriculture and Life Sciences, Chonnam National University, Gwangju 61186, Republic of Korea; qkrcjfwn3@naver.com (C.P.); sg90813@naver.com (S.J.)

**Keywords:** rat, deoxynivalenol, apoptosis, fibrosis, microbiota, metabolite

## Abstract

Mycotoxin-contaminated feed or food can affect physiological responses and cause illnesses in humans and animals. In this study, we evaluated the effects of deoxynivalenol (DON) toxicity on the growth performance, blood biochemistry, histology, microbiome, and metabolism of rats fed with different toxin concentrations. After 1 week of acclimatization, seven-week-old male rats received 0.9% saline as a control, 0.02 mg/kg DON as T1, and 0.2 mg/kg DON as T2 via oral gavage for 4 weeks. The final body weight of the T2 group was significantly lower than that of the control and T1; however, the average daily gain, feed intake, and feed conversion ratio did not differ. Fibrosis and apoptosis were observed in various tissues as DON concentration increased. Creatinine and alkaline phosphatase levels were significantly lower in the DON-treated group than in the control. Firmicutes and Desulfobacterota phyla dominated the cecum, whereas those in the feces were Proteobacteria and Bacteroidetes. Metabolomic profiling showed phenylalanine, tyrosine, and tryptophan biosynthesis as the most prominent pathways. Overall, our results suggest that low-dose and short-term DON exposure can trigger several adverse effects in rats. Dietary toxicants in rats may explain the physiological effects associated with the metabolism commonly reported in animals.

## Simple Summary

Mycotoxins are secondary metabolites produced by fungi and are commonly found in feed and food worldwide. Specifically, deoxynivalenol (DON) is a common mycotoxin found in cereal grains that can negatively affect food safety and human and animal health. This in vivo study using rats evaluated the harmful effects of DON in animal feed. Our results showed that the impact of DON exposure on growth characteristics, histological alterations, microbiota composition, and metabolomics profiles was more pronounced in the DON-contaminated rat group than in the control group. Rats fed DON showed growth retardation and histological changes, including fibrosis and apoptosis in specific organs. Moreover, DON-contaminated rats showed significantly altered levels of metabolic biomarkers, including phenylalanine and tryptophan, in various tissues such as blood, liver, and cecum. Furthermore, this study builds upon previous research and provides insights into the influence of microbiota composition on DON. Our findings suggest the risks associated with DON in feed, and this study significantly contributes to the existing literature.

## 1. Introduction

Mycotoxins, common contaminants in food and feed worldwide, are secondary metabolites produced by fungi [1]. Deoxynivalenol (DON), a common mycotoxin found in cereals, is a B-type trichothecene produced by Fusarium species [2,3]. Its effects on protein synthesis and immune responses are significant because they threaten food safety and human and animal health [4].

Given the extensive food contamination caused by DON, it significantly affects public health [5]. The European Food Safety Authority (EFSA) reported that DON was detected in 43.5% of food samples and 75.2% of feed samples collected in the EU [6]. Furthermore, 95% of 494 feed samples in Korea were contaminated with DON [7]. Moreover, 94% of 707 feed samples from the United States contained DON [8]. In this context, the United States Food and Drug Administration (FDA) has established a maximum concentration of ˂1 mg/kg for DON. Similarly, the European Commission has established a limit of ˂0.9 mg/kg [9,10]. Growing evidence links DON-associated food contamination to various adverse effects on human and animal health [1].

Several studies have demonstrated that ingesting feed contaminated with DON causes vomiting- and anorexia-induced growth retardation in animals, leading to significant economic losses in livestock production [11,12]. In addition, DON affects systemic immune responses and alters blood biochemistry in animals [13]. Consequently, the intestinal barrier is compromised, which in turn reduces nutrient absorption and utilization, and disrupts the gut microbiota [14,15,16]. Furthermore, DON induces reactive oxygen species (ROS), resulting in lipid peroxidation and the modulation of intracellular antioxidant systems [17]. These effects contribute to histological changes, such as lymphodepletion, apoptosis, and fibrosis, among the major adverse consequences of DON exposure in animals [1,18]. In particular, DON can cause liver damage by interfering with protein synthesis and causing excessive generation of ROS. This may be associated with reduced ALKP activity [16,18]. The main target of DON is the intestinal epithelial cells. The apoptosis observed in small intestinal cells in this study may be closely related to oxidative stress [19]. DON induces excessive ROS production and significantly reduces the concentration and function of antioxidant enzymes. This leads to cellular oxidative stress [20]. The gastrointestinal tract, colonized by a complex bacterial community, is the first site of DON toxicity after ingestion of contaminated feed. By preventing pathogen colonization and the production of fermentation products, the gut microbiota plays an important role in protective, nutritional and immunological functions.

The effects of DON were dependent on species, concentration, duration of ingestion, purity, and method of administration; however, the effects of low-dose exposures have not been well studied. Therefore, we designed our experimental groups to observe in vivo changes at short-term, low concentrations. Rats fed diets containing 0.25, 0.5, or 1.0 mg/kg bw DON for 6 weeks were observed to dilate the renal pelvis and urinary bladder [21]. Male Wistar rats fed diets contaminated with different doses of DON (0, 0.2, 0.75, 1.75, and 2 mg/kg) for 42 days had no change in body weight but had reduced ganglion area [22]. Rats were administered 0, 0.03, 0.1, 0.3, 1, or 3 mg/kg/day DON once daily by gavage from gestation day 6 to postnatal day 27. DON had no effect on maternal body weight or feed consumption at any dose. A dose of 3 mg/kg/day resulted in mild toxicity, which was manifested as decreased body weight in the offspring [23].

Overall, susceptibility to food contamination by DON is highest in swine, followed by mice, rats, poultry, and ruminants [24]. Notably, studies to determine the toxicity of high doses of mycotoxins are typically conducted in vivo using murine models [1]. Animal models are important for understanding interactions with whole biological systems because they can be studied across the life span. To assess the risks posed by DON in animal feed, it is important to determine the DON content of the feed samples. Therefore, our objective in this study was to investigate the dose-dependent effects of the oral administration of DON at different concentrations in rats on growth performance, blood biochemistry, metabolism, and intestinal microbiota.

## 2. Materials and Methods

### 2.1. Ethics Statement

All the experimental procedures were reviewed and approved by the Institutional Animal Care and Use Committee of the National Institute of Animal Science, Korea (No. NIAS-2022-0546).

### 2.2. Animal Care and Design

Male Sprague–Dawley (SD) rats weighing 140–150 g were obtained from Koatech (Pyeongtaek, Korea). Fifteen SD rats were housed in individual cages (277 mm × 423 mm × 194 mm). During the experimental period, the housing conditions, including acclimatization, were as follows: (1) light-dark cycle of 12:12 h, (2) constant temperature (23 ± 2 °C), and (3) relative humidity (55 ± 5%) according to the growth period. The animals were acclimated to laboratory conditions for one week prior to the experimental period. The rats were divided into three groups: (1) control (n = 5) fed a basal diet; (2) T1 group (n = 5), basal diet + 0.02 mg DON/L; and (3) T2 group (n = 5), basal diet + 0.2 mg DON/L. Many previous studies have shown that high DON intakes affect growth. However, we designed the experimental groups to measure in vivo changes at short-term and low concentrations based on previous studies and our other experimental results. The animals were administered 0.9% saline or DON mixed with 0.9% saline daily for 28 days via oral gavage. Animals had access to food and water ad libitum throughout the study period. DON (TripleBond, Guelph, ON, Canada) was thoroughly mixed with an organic solvent (95% ethyl alcohol; Lab Alley, Austin, TX, USA) according to the experimental concentrations. All animals were anesthetized using CO_2_. In addition, blood and tissues, including the liver, kidney, muscle, abdominal fat, and jejunum, were rapidly collected. The collected tissues, cecum contents, and feces were frozen immediately in liquid nitrogen and stored at −80 °C (UniFreez U500, Daihan Scientific Co., Wonju, Republic of Korea). For histological analysis, the tissues were fixed in 10% neutral-buffered formalin (NBF; Sigma-Aldrich, St. Louis, MO, USA). The following formulae were employed to calculate the average daily gain (ADG), average daily feed intake (ADFI), and feed conversion ratio (FCR):

ADG = (final weight − initial weight)/age (d)

ADFI = amount of feed provided − amount of feed remaining

FCR = feed intake/average daily gain

### 2.3. Blood Biochemical Analysis

Blood samples were collected via cardiac puncture using a Vacutainer tube without anticoagulants. Serum was taken by centrifugation (700× *g* for 15 min at 4 °C) (VS-550, Vision Scientific Co., Daejeon, Republic of Korea) and then stored at −80 °C (UniFreez U500, Daihan Scientific Co., Wonju, Republic of Korea). Fifteen parameters were analyzed, including glucose (GLU), creatine (CREA), blood urea nitrogen (BUN), phosphate (PHOS), calcium (CA), total protein (TP), albumin (ALB), globulin (GLOB), alanine aminotransferase (ALT), alkaline phosphatase (ALKP), total bilirubin (TBIL), cholesterol (CHOL), amylase (AMYL), and lipase (LIPA). The parameters were determined using a VetTest chemistry analyzer (IDEXX, Westbrook, ME, USA) according to the manufacturer’s instructions.

### 2.4. Histological Analysis

The study of apoptosis and fibrosis induced by DON may facilitate the understanding of the processes of tissue damage and repair, including the regulation of inflammatory responses, and provide insights into disease prevention. Liver, kidney, muscle, abdominal fat, and jejunum portions (0.5 cm × 0.5 cm) were collected from all rats. Each sample was fixed in 10% NBF and embedded in paraffin. The following brief procedure was employed: tissue samples were fixed in 10% NBF, dehydrated from 70–100% ethanol (EtOH, Sigma Aldrich, Steinheim, Germany), embedded in xylene (Sigma Aldrich, Germany), sectioned (5 μm thick), and heated (45 °C) for 3 h on a slide warmer (77 Slide Warmer, Fisher Scientific, Waltham, MA, USA). For staining, the slides were deparaffinized with xylene, rehydrated from 100–70% EtOH, and washed with distilled water. Sections for fibrosis and apoptosis were stained using Masson’s trichrome staining reagent and an in situ Cell Death Detection Kit (POD) in accordance with the manufacturer’s instructions. Stained sections were examined under a microscope (Micrometrics; Nikon ECLIPSE E200, Tokyo, Japan) at 200× magnification.

### 2.5. Microbial Sequencing and Data Analysis

Microbial DNA was extracted from the cecum and fecal contents using the bead-beating plus column method with a QIAamp DNA kit (Qiagen, Hilden, Germany) following the manufacturer’s protocol [25]. 1% agarose gel electrophoresis and a microplate reader (Infinite M NANO, Tecan, Republic of Korea) were used to analyze the quality and quantity of extracted DNA. The 16S rRNA genes were amplified using 16S V3-V4 primers. The primer sequences were as follows: forward primer 341F (5′-CCTACGGGNGGCWGCAG-3′) and reverse primer 805R (5′-GACTACHVGGGTATCTAATCC-3′) [26]. Libraries were sequenced on a MiSeq platform (Illumina, San Diego, CA, USA). The resulting 16S amplification sequences (Macrogen, Daejeon, Republic of Korea) were cleaned and analyzed using Quantitative Insights into Microbial Ecology (QIIME 2, Ver. 2021.8) [27] and MicroBiomeAnalyst. Furthermore, a plugin designated DADA2 was utilized for the removal of adapters and chimeric sequences and for quality filtering, denoising, and merging operations [28]. Subsequent analysis of ASVs assessed the microbial diversity and taxonomy. Amplicons of interest were taxonomically classified according to the SILVA taxonomy database (version 138). Alpha diversity indices, including observed amplicons, Chao1, Shannon’s index, and Simpson’s index, were evaluated based on amplicon sequence variant biological observation matrix ASV tables. Additionally, the beta diversity of the fecal microbiota among the four treatment groups was analyzed using principal coordinate analysis (PCoA) on Bray-Curtis dissimilarity matrices. 

### 2.6. Metabolites Preparation and Analysis of Blood, Cecum, Feces, Kidney, and Liver Contaminated with Deoxynivalenol

One hundred microliters of serum were combined with 400 µL of cold acetone (Merck Millipore), placed in a refrigerator (MPR-N250FH, PHCBI Co., Seoul, Republic of Korea), and shaken (Rotamix-SLRM1, Seoulin Bioscience Co., Seoul, Republic of Korea) for 1 h. The 400 µL supernatant was decanted, placed in a speed vacuum until completely dried, followed by dissolution in 100 µL of 20% methanol (internal standard—terfenadine; Merck Millipore). The resulting solution was analyzed using UPLC-Q-TOF MS (Waters, Milford, MA, USA). The liver, cecum, urine, and fecal samples were lyophilized and extracted as follows: the liver, cecum, and fecal samples were dissolved in 80% methanol with an internal standard (terfenadine, Merck Millipore), and urine was dissolved in 20% methanol. Upon completion of metabolomic analysis of each rat sample, the samples were cross-mixed for further analysis. The sample extracts were injected onto an Acquity UPLC BEH C18 column (2.1 mm × 100 mm, 1.7 um; Waters) using a mobile phase consisting of water with 0.1% formic acid (A) and acetonitrile (ACN) with 0.1% formic acid (B) at a flow rate of 0.35 mL/min, with analysis time of 12 min for blood, liver, cecum, feces, and urine and 16 min for blood and urine at a column temperature of 40 °C. The elements that passed through the column were analyzed using Q-TOF MS in the positive electrospray ionization (ESI) mode. The TOF-MS data were scanned between 100 and 1500 *m*/*z* with a scan time of 0.2 s. The capillary and sample cone voltages were 3 V and 40 V, respectively. The de-solvation flow rate was 800 L/h, while the de-solvation temperature was 300 °C, and the source temperature was 100 °C. Leucine-enkephalin ([M + H] = 556.2771) was used as a reference compound because of its low mass and was analyzed every 10 s. A quality control (QC) sample, prepared by mixing all samples, was analyzed every 10 analyses. The MS/MS spectra were obtained using a collision energy ramp (10–45 eV) at *m*/*z* 50–1500. Mass spectrometry data were processed using MarkerLynx 4.1 software (Waters), which included the calculation of the mass-to-charge ratio (*m*/*z*), retention time, and ion intensity. The acquisition, normalization, and alignment of the LC-MS data obtained using UPLC-Q-TOF MS were performed using the MarkerLynx program. Peak-to-peak baseline noise, noise elimination, peak width (5% height of 1 s), and intensity threshold (10,000) were used to identify the peaks, whereas a mass window (0.05 Da) and retention time window (0.2 min) were used to align the metabolite data. All data were normalized to standard values. Metabolites were identified using a combination of ChemSpider (www.chemspider.com, accessed on 9 August 2019), human metabolome databases (www.hmdb.ca, accessed on 7 January 2022), METLIN database (https://metlin.scripps.edu, accessed on 24 August 2020), literature, and standards.

### 2.7. Statistical Analysis

Statistical analyses of the LC-MS data were conducted using SIMCA-P+ version 12.0.1 (Umetrics, Umeå, Sweden). Partial least squares discriminant analysis (PLS-DA) was used to visualize the results, which were evaluated using R2X, R2Y, Q2, and permutation tests. A permutation test was performed to cross-validate the PLS-DA results. Furthermore, the relative abundance of metabolites was analyzed by one-way analysis of variance (ANOVA) with Duncan’s test (*p* < 0.05) using SPSS 17.0 (SPSS Inc., Chicago, IL, USA). Heatmaps of the identified compounds were generated using R with the gplots package. They were generated on a red-white-blue color scale based on the z-score, where red indicated a decrease in metabolite content, blue indicated an increase, and white indicated no change. The differential abundance of taxa among the three treatment groups was analyzed using the linear discriminant analysis (LDA) effect size (LEfSe) (LDA score > 3). Growth performance, biochemical analyses, and alpha diversity indices were compared among the three treatment groups using analysis of variance (ANOVA) with Tukey’s test using Prism statistical software (ver. 9.5.1, GraphPad Software, San Diego, CA, USA). We used linear regression to analyze the relationship between final body weight and biochemical parameters and metabolites. Beta diversity and functional genetic profiles were compared among the four treatment groups using permutational multivariate analysis of variance (PERMANOVA) with PAST3 and 9999 random permutations. The results are expressed in terms of mean and standard error of the mean (SEM). Statistical significance was set at *p* < 0.05, indicating a significant difference between the control and treatment groups.

## 3. Results

### 3.1. Growth Performance

**Figure 1:**
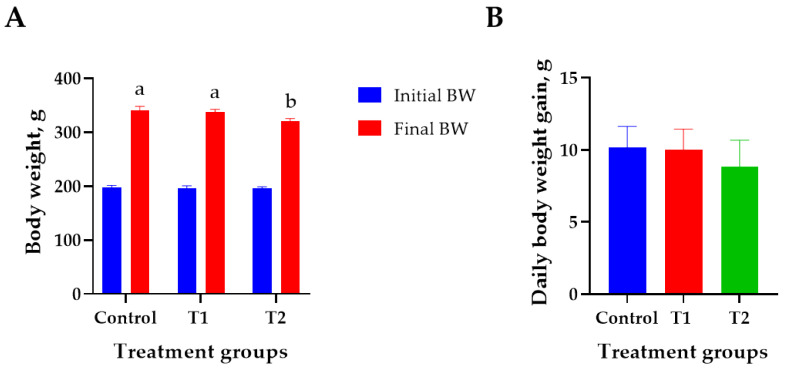

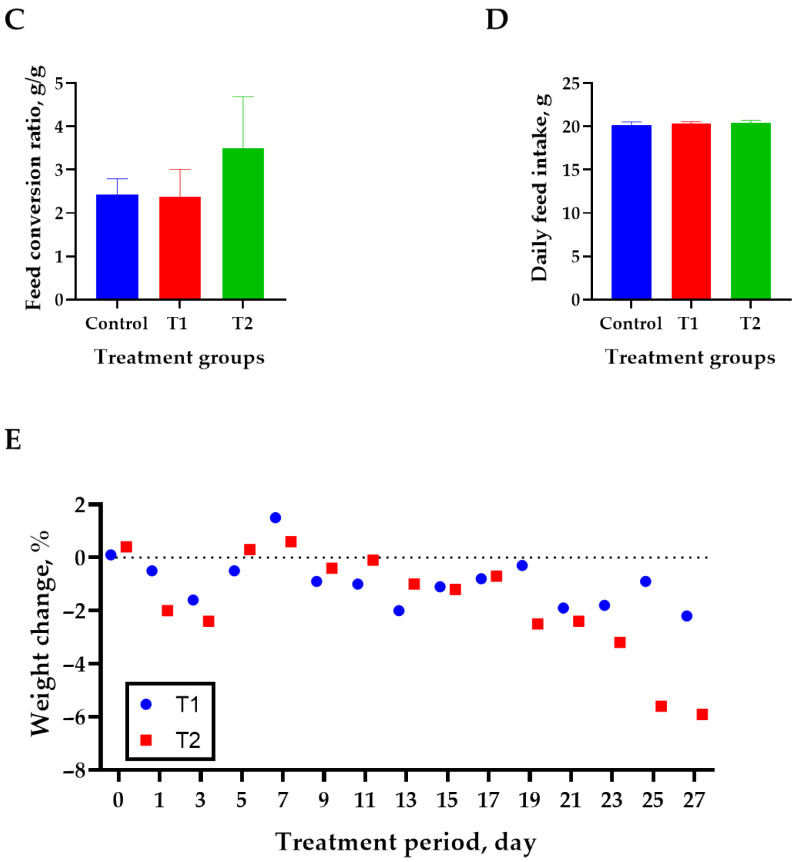
Effects of DON ingestion on growth performance in rats. (**A**) Initial body weight of 6 −week-old rats and final body weight after 4 weeks of DON ingestion. (**B**) Average daily weight gain for 4 weeks after the start of the experiment. (**C**) Feed conversion ratio for 4 weeks after the start of the experiment. (**D**) Average daily feed intake for 4 weeks after the start of the experiment. (**E**) Proportion of rats’ body weight change in T1 and T2 groups for 4 weeks after the start of the experiment. Treatment group: Control, basal diet; T1, basal diet + 0.02 mg/kg DON; T2, basal diet + 0.2 mg/kg DON. ^a,b^ Different superscript letters indicate that variables within a row differed significantly (*p* < 0.05). Weight change (%) = (body weight of control − body weight of treatment group/body weight of control) × 100.

The effects of DON treatment on the growth performance of 6-week-old rats over 28 days are shown in Figure 1. The initial body weight (194 ± 2.8 g) did not differ significantly among the treatment groups. The T2 (0.2 mg/L) group showed the lowest final body weight (320 ± 4.2 g) than that of the control (340 ± 6.9 g) on day 27 (*p* < 0.05, Figure 1A). However, the ADG, FCR, and ADFI did not differ significantly among the treatment groups (Figure 1B–D). Weight change during the study was reduced by 0.9% at T1 and 5.9% at T2 compared to the control (Figure 1E).

### 3.2. Blood Biochemistry

The effects of DON treatment on 17 blood biochemical parameters in rats for 28 days are presented (Table 1). The levels of CREA and ALKP in the blood of DON-treated rats were significantly lower than those in the control group (*p* < 0.05). Moreover, an elevated BUN-to-CREA ratio was detected in the DON-treated rats compared to the control; however, this was not statistically significant.

### 3.3. Histology

**Figure 2:**
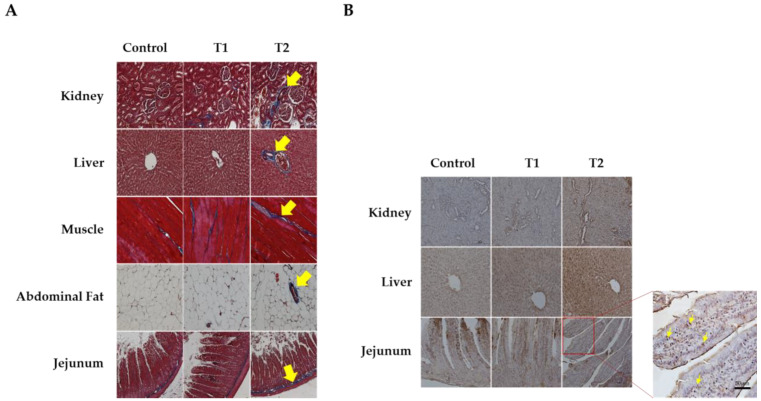
Images of fibrosis and apoptosis in DON-contaminated rats after 4 weeks from the start of the experiment. (**A**) Representative figure of fibrosis observed in the kidney, liver, muscle, adipose tissue, and jejunum by Masson’s trichrome staining according to the DON level. (**B**) Representative apoptotic figures were observed in the kidney, liver, and jejunum by TUNEL staining according to DON levels. Treatment group: Control, basal diet; T1, basal diet + 0.02 mg/kg DON; T2, basal diet + 0.2 mg/kg DON. The arrows indicate apoptosis and fibrosis. Observations were performed at 200× magnification.

We conducted Masson’s trichrome staining to analyze histological changes in the kidney, liver, muscle, abdominal fat, and jejunum, as shown in Figure 2A. The kidney tissue stained blue around the glomeruli and tubules, indicating an increase in fibrosis in response to DON. The central venous vessels and bile ducts of the liver showed a significant increase in fibrosis. Furthermore, increased fibrosis of the endomysium surrounding the individual muscle fibers is associated with increased DON levels in muscle tissue. In addition, abdominal fat was stained blue in the connective tissue and veins. Fibrosis was observed in the intramuscular mucosa adjacent to the submucosa of the jejunal tissue. The data indicated that all five tissues showed evidence of fibrosis as DON levels increased, particularly in the T2 group.

Figure 2B shows the results of TUNEL staining for apoptosis in the kidney, liver, and jejunum. We observed obvious signs of apoptosis in the apical regions of the jejunum villi, renal cells, and hepatocytes. The results indicated a dose-dependent increase in apoptosis with increasing DON levels in tissue cells, and the T2 group significantly increased in TUNEL-positive staining compared to the control.

### 3.4. Metabolomic Profiling in Cecal and Fecal Tissues Contaminated with Deoxynivalenol

**Figure 3:**
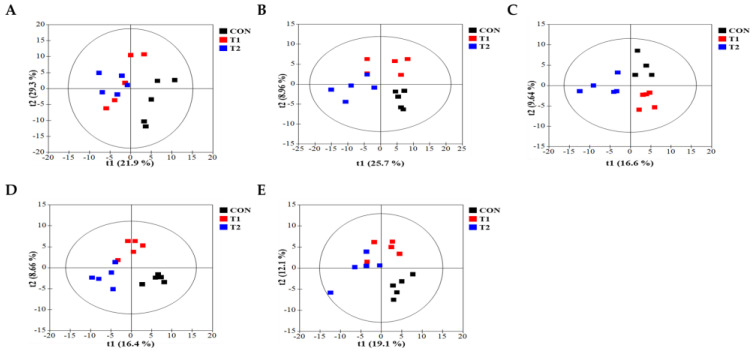

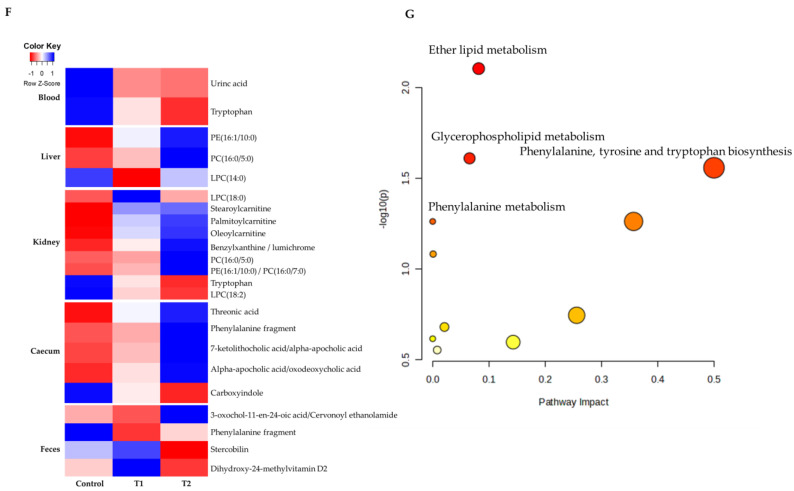
Metabolite profiling of blood, cecum, feces, kidney, and liver from DON−contaminated rats. (**A**–**E**) PLS-DA score plot of blood (**A**), cecum (**B**), feces (**C**), kidney (**D**), and liver (**E**). (**F**) Heatmap of the changes in metabolites related to DON levels. The blue color represents an increasing trend, and red represents a declining trend. (**G**) It represents the results of metabolic pathways in different DON treatment groups. Variations in score plots were defined using a 95% confidence interval. The heatmap shows the significantly different data visualization of multiple parameters. Treatment groups: Control, basal diet; T1, basal diet + 0.02 mg/kg DON; T2, basal diet + 0.2 mg/kg DON.

**Figure 4:**
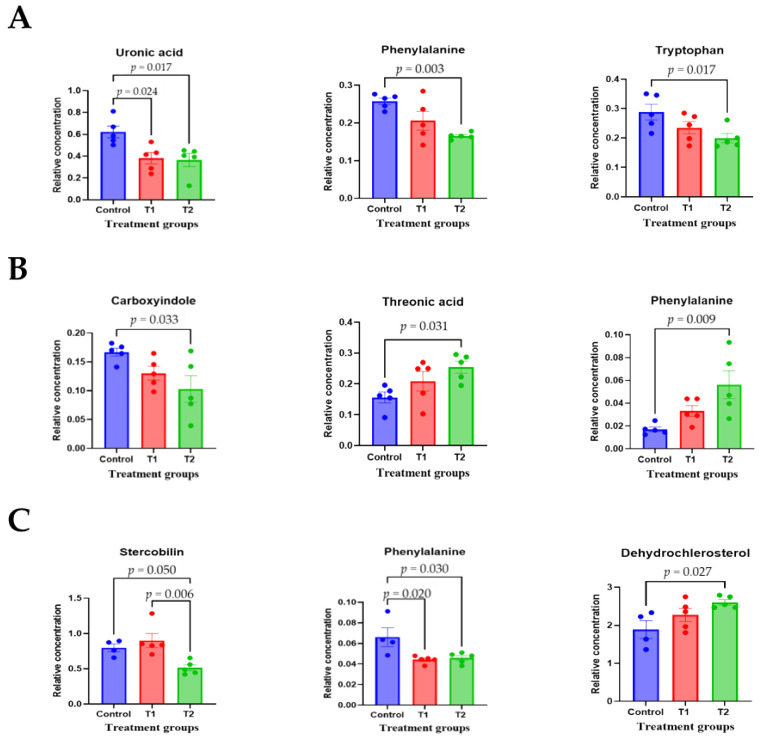

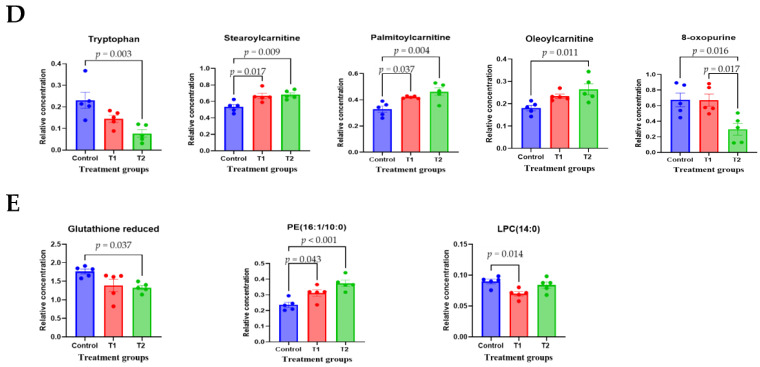
The scatter and bar graph of important variables in the projection of metabolites in five different tissues. (**A**–**E**) Blood, cecum, feces, kidney, and liver, respectively. Metabolites in five different tissues were significantly different in the ANOVA model based on mean comparison using Tukey’s test. Treatment groups: Control, basal diet; T1, basal diet + 0.02 mg/kg DON; T2, basal diet + 0.2 mg/kg DON.

To comprehensively understand DON’s effect on metabolism, we characterized the metabolites in different rat tissues using liquid chromatography-mass spectrometry. Partial least squares discriminant analysis (PLS-DA) showed a distinct separation of metabolites in the DON-treated groups compared to the control (Figure 3A–E). DON-induced metabolites were separated between the control and treatment groups in all tissues. Particularly, the cecum, feces, and kidneys were different in all groups. As shown in Figure 3F, a heat map was generated by hierarchical Pearson clustering of selected blood, liver, kidney, cecum, and fecal metabolites. The heat map result indicated that DON shows changes, including amino acids, lipids, and carbohydrates in blood and tissues (*p* < 0.05). Some metabolites in blood and feces were decreased due to DON, whereas most increased levels were observed in the liver, kidney, and cecum. The results showed that the control and the two DON-treated groups differed significantly (FDR rate, *p* < 0.05). Metabolites without clear KEGG structures were excluded. As illustrated in Figure 3G, the DON-treated group predominantly showed alterations in ether lipid metabolism, followed by glycolipid, phenylalanine, tyrosine, and tryptophan biosynthesis, and phenylalanine metabolism. We performed further statistical analyses of DON contamination in rats to identify the potential tissue-specific biomarkers. Metabolites of all tissues, such as the blood, kidney, liver, feces, and cecum, were significantly changed based on VIP scores *>* 1.0 and *p <* 0.05 (Figure 4). Furthermore, significant differences in the metabolite profiles were observed between the DON and control groups. The most significantly differentiating compounds between the control and DON groups were uronic acid, phenylalanine, tryptophan, carbolyindole, threonic acid, stercobilin, dehydrochlorosterol, stearolcartinine, palmitoylcanitine, oleoylcarnitine, 8-oxopurine, reduced glutathione, PE (16:1/10:0), and LPC (14:0). Among the candidate metabolites, phenylalanine was decreased in the blood and feces of the T2 group, whereas it was increased in the cecum. Similarly, a significant decrease in tryptophan levels was observed in both the blood and kidneys. Seven of the significant metabolites were increasedm and nine were decreased in the T2 group compared to the control group.

### 3.5. Microbial Composition of Cecal and Fecal Tissues Contaminated with Deoxynivalenol

**Figure 5:**
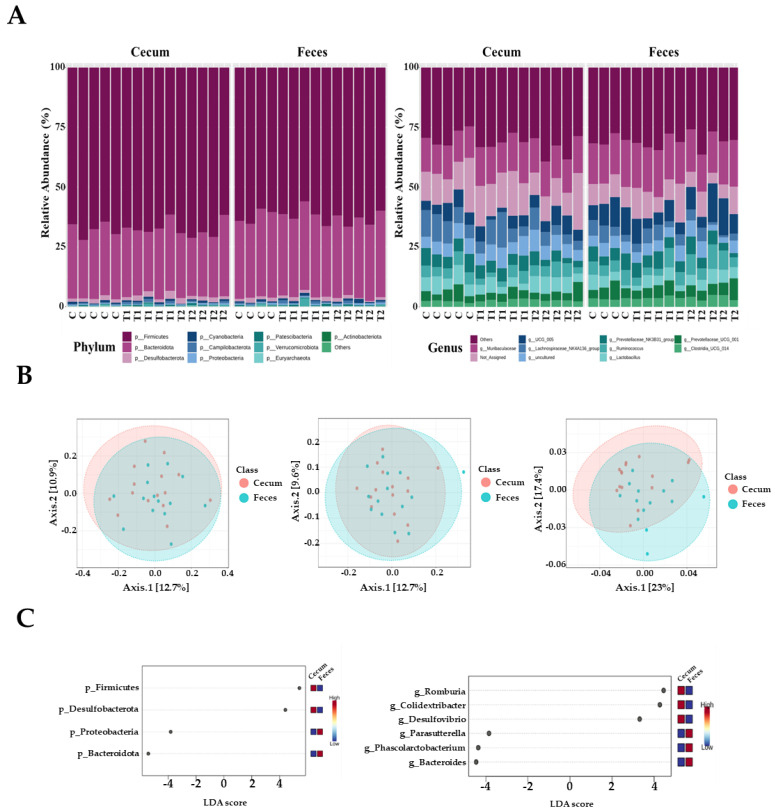
Microbiota profiles from the cecum and feces contents of the three dietary treatment groups at the phylum and genus levels. (**A**) Bars represent the relative abundance (%) of each phylum (left) and genus (right) level detected per sample. (**B**) Microbiota beta diversity indices between the two different tissues, including the Bray−Curtis index, unweighted UniFrac distance, and weighted UniFrac distance. (**C**) Graphical representation of linear discriminant analysis (LDA) effect size (LEfSe) between the two different tissues. The horizontal bar represents the log10−transformed LDA score. The bacterial taxa were statistically significant (*p* < 0.05) in terms of relative abundance. Treatment groups: Control, basal diet; T1, basal diet + 0.02 mg/kg DON; T2, basal diet + 0.2 mg/kg DON.

In total, 1,469,468 high-quality sequences were obtained from 29 cecal and fecal samples, with 15 samples derived from the cecum and 14 from feces. The three dominant phyla were Firmicutes, Bacteroidetes, and Desulfobacter. Firmicutes were more prevalent, accounting for 68.5% and 62.9% of all sequences in the cecum and feces, respectively (Figure 5A). Bacteroidetes were next in dominance, representing 27.1% and 32.3% of all sequences in the cecum and feces, respectively. Desulfobacterota constituted 1.8% and 1.3% of the total sequences in the cecum and feces, respectively. The remaining phyla collectively accounted for less than one percent of all sequences. However, the most dominant genus in the cecal samples was *Muribaculaceae*, accounting for 7.9% of all sequences. These were followed by UCG-005 (5.3% and 9.3%), *Lactobacillus* (5.6% and 4.5%), and *Ruminococcus* (3.8% and 4.9%, respectively). At the genus level in fecal samples, *Muribaculaceae* was the most dominant, accounting for 9.6% of all sequences. This was followed by UCG-005 (9.3%), *Ruminococcus* (4.9%), and *Lactobacillus* spp. (4.5%). 

The observed amplicon sequence variants (ASVs) and Chao1, ACE, Shannon, and Simpson indices were not significantly different between the two tissue groups. Beta diversity analysis based on Bray–Curtis dissimilarity matrices and unweighted UniFrac distance showed no significant differences between the two tissue samples. However, we observed that the two samples differed significantly when the weighted UniFrac distance was used (*p* < 0.05) (Figure 5B). At the phylum level, LEfSe analysis showed that Firmicutes and Desulfobacterota were more prevalent in the cecal tissue group than in fecal samples; however, Bacteroidetes and Proteobacteria were more abundant in fecal samples than in cecal samples (Figure 5C). At the genus level, *Romboutsia*, *Colidextribacter*, and *Desulfovibrio* were more abundant in the cecum than in the feces. Furthermore, *Parasutterella*, *Phascolarctobacterium*, and *Bacteroides* were more prevalent in the fecal samples than in the cecal samples (Figure 5C).

**Figure 6:**
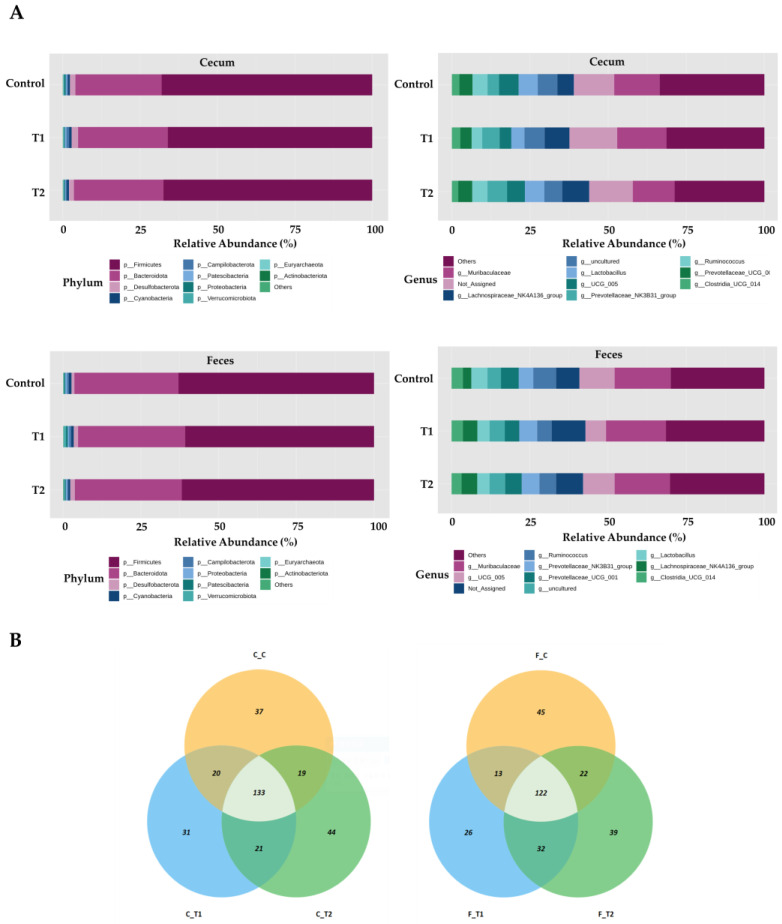
Differentially abundant phyla and genera in the cecum and feces among the DON-treated groups. (**A**) Bars represent the relative abundance (%) of each phylum (upper-left side) and genus (upper-right side) in the cecum sample, and each phylum (lower-left side) and genus (lower-right side) detected in the feces samples. (**B**) Venn diagrams showing the core microbiome of genera, shared in the cecum (left) and feces (right) samples. Treatment groups: Control, basal diet; T1, basal diet + 0.02 mg/kg DON; T2, basal diet + 0.2 mg/kg DON.

Overall, the most prevalent phylum was Firmicutes, which accounted for 68.8%, 67.6%, and 69.4% of the total sequences in the control, T1, and T2 groups of cecum tissue, respectively (Figure 6A). Bacteroidetes were next in dominance, representing 27.6%, 27.3%, and 26.5% of all sequences in the control, T1, and T2 cecum tissues, respectively. At the genus level in cecal samples, *Muribaculaceae* was the most dominant, representing 7.3%, 8.8%, and 7.9% of all sequences in the control, T1, and T2 cecum tissues, respectively. Subsequently, *Lactobacillus* was identified with a prevalence of 6.3% in the control group, 4.3% in T1, and 6.2% in T2. Firmicutes were the most dominant in fecal samples, representing 62.9%, 62.8%, and 65.0% of all sequences in the control, T1, and T2 fecal samples, respectively. This was followed by Bacteroidetes, which represented 33.3%, 32.4%, and 31.3% of all sequences in control, T1, and T2 fecal tissues, respectively. At the genus level in fecal samples, *Muribaculaceae* was the most dominant, accounting for 9.4%, 10.5%, and 9.0% of all sequences in the control, T1, and T2 of the fecal samples, respectively. UCG-005 was the next most abundant genus, accounting for 9.8%, 7.0%, and 11.2% of all sequences in the control, T1, and T2 fecal samples, respectively. The OTUs in the ileum and feces were mapped using Venn diagrams, which revealed a comparable number of overlaps in each tissue type. (Figure 6B).

### 3.6. Correlation Analysis of Biochemical Parameters and Metabolites

**Figure 7:**
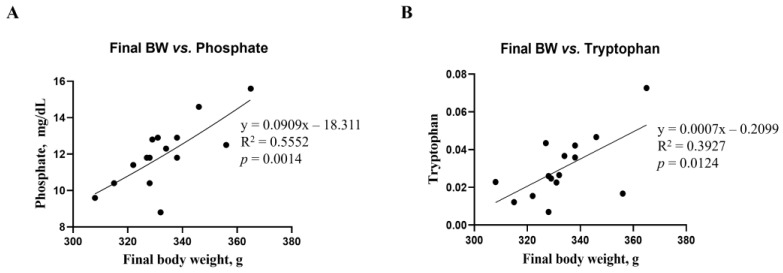
Simple linear regression analysis showing the association between final body weight and biochemical parameters, as well as kidney metabolites, of the DON-treated groups. (**A**) Phosphate parameter in blood. (**B**) Tryptophan in kidney metabolites. Treatment groups: Control, basal diet; T1, basal diet + 0.02 mg/kg DON; T2, basal diet + 0.2 mg/kg DON. The correlation coefficient and *p*-value were calculated using GraphPad Prism software.

Correlations between final body weight and blood biochemical parameters, as well as metabolites, were analyzed using a linear regression model (Figure 7). The correlations between final body weight and blood biochemical parameters and metabolites were consistent. Phosphate among the biochemical parameters (R^2^ = 0.5552, *p* = 0.0014, Figure 7A) and tryptophan among renal metabolites (R^2^ = 0.3927, *p* = 0.0124, Figure 7B) showed significant differences from the mean final body weight. However, no differences in microbiota compositions were found in this study.

## 4. Discussion

The present study examined the effect of DON on growth performance, blood biochemistry, histology, metabolites, and microbiota composition in rats over four weeks. Furthermore, the potential relationship between body weight and individual results was investigated. Rats ingesting 0.2 mg/kg of DON exhibited reduced growth performance and changes in the biochemical properties of blood and metabolism. Furthermore, our results showed that such ingestion leads to crucial histological changes, including fibrosis and apoptosis, in specific organs of the rats. Therefore, our findings suggest that DON concentrations below the residual limit may cause adverse health effects in rats.

Reportedly, DON has negatively affected the growth performance of animals in previous studies [17,29,30]. Growth retardation is a major symptom of DON toxicity, and its effects can cause severe anorexigenic effects and vomiting, which can reduce feed intake in animals [31]. These adverse effects in animals are primarily determined by DON concentrations [32]. In our study, the oral administration of DON at a low dose of 0.02 mg/kg in rats did not significantly affect growth performance; however, a dose of 0.2 mg/kg resulted in the lowest final body weight than that of the control for the short-term period. Male rats fed DON showed a 12% decrease in final body weight [17], which is consistent with our results. However, our results showed that DON did not affect the ADFI for 4 weeks, suggesting that the observed weight loss was due to the multifaceted effects of DON. DON reduces nutrient absorption and utilization by damaging the intestinal barrier [14,15]. Additionally, DON can interfere with the normal metabolic function of organs, leading to inefficient use of the consumed nutrients [33]. Therefore, it is necessary to understand the effects of DON on animal growth.

The effects of DON treatment on the blood biochemistry of the rats were limited. The CREA concentrations were significantly affected by DON treatment. In this study, the CREA and BUN/CREA ratios were the main parameters reflecting the degree of kidney damage [34,35]. DON causes oxidative stress in kidney cells and kidney dysfunction, which may be related to our results [34]. The decrease in CREA levels in rats administered DON in our study may indicate degenerative kidney function [36]. In addition, previous studies report that a higher BUN/CREA ratio is associated with various health problems, such as heart failure and chronic kidney disease [37]. Furthermore, our results showed a decrease in ALKP activity following DON treatment, similar to the findings of [17]. Blood ALKP levels are the most reliable for assessing liver damage [38]. DON can severely damage the liver because it is the primary organ that detoxifies and metabolizes mycotoxins in the body [39]. DON can cause liver damage by interfering with protein synthesis and causing excessive generation of reactive oxygen species (ROS), which may be associated with decreased ALKP activity [17,24,40]. Blood parameters are affected by DON. Changes in blood parameters such as CREA and ALKP can indicate underlying health problems such as kidney and liver damage. These may also affect body weight. DON exposure can cause kidney and liver damage, which can affect these blood parameters and reduce body weight. High CREA typically indicates kidney problems, while high ALKP indicates liver or bone problems. However, in this study, DON caused weight loss and kidney and liver damage, but reduced CREA and ALKP levels.

From the results of this study, we observed a dose-dependent effect on histological changes, including apoptosis and fibrosis, in multiple organs such as the kidney, liver, muscle, adipose tissue, and small intestine. The principal target of DON is the intestinal epithelial cells, and the apoptosis observed in small intestinal cells in this study may be closely related to oxidative stress [19]. As discussed previously, DON induces excessive ROS production and significantly reduces the concentration and function of antioxidant enzymes, resulting in cellular oxidative stress [20]. A study using porcine models revealed that DON-induced oxidative stress increases the expression of genes associated with inflammatory processes and apoptosis in the epithelial cells of the intestine [19]. Furthermore, the oxidative stress induced by DON can cause fibrosis [41]. This phenomenon is defined by the excessive accumulation of matrix and connective tissue components, which can affect various organs [42]. Oxidative stress promotes the expression of target fibrotic genes, including tumor growth factor beta 1. This interaction induces fibrosis through extracellular matrix accumulation [43,44]. Furthermore, damaged cells from oxidative stress can initiate an inflammatory response, and the sustained activation of this response can lead to tissue fibrosis [43,44,45]. Apoptosis and fibrosis in the liver, kidney, and gastrointestinal tract have been linked to chronic diseases such as renal failure and cirrhosis. Thus, DON can alter nutrient absorption, feed efficiency, and inflammation, resulting in weight loss.

The microbiota significantly influence the productivity, health, and well-being of animals. Previous studies have shown that the microbiota can be influenced by several factors, including dietary intake, host species, breed, geographical location, and seasonality. These factors also affect the rat’s microbiota, particularly through diet. Consequently, we investigated the effects of different diets on the composition of the cecal and fecal microbiota in rats exposed to DON. 

Ingesting feed contaminated with DON represents an initial exposure to the intestinal tract, which is the primary barrier against contaminants, chemicals, and pathogens [46,47]. The negative effects of DON on the gastrointestinal microbiome are evident, including impaired intestinal integrity and function. The location of DON within the tract also affects microbial composition and abundance [48,49]. We observed that Firmicutes was the most dominant phylum, and Muribaculaceae was the most dominant genus, with no significant differences among the treatment groups. In contrast, an increased Coprococcus abundance in rats fed DON (60 and 120 μg/kg body weight) has been reported [50]. Another study found increased Bacteroides and Prevotella abundance after administering 100 µg/kg body weight of DON orally for 4 weeks [4]. The results differed due to variations in age, DON levels, toxin status, feeding duration, climate, and dietary composition [13]. Furthermore, we found differences in the relative abundance between the cecum and feces at both the phylum and genus levels. The gastrointestinal tract varied significantly in nutritional and chemical composition, temperature, and pH depending on its location [51]. Therefore, our results may be due to regional differences in the gut microbial composition influenced by the functional diversity of feces and different segments of the gastrointestinal tract. In this study, the dominant phyla in the cecum were Firmicutes and Desulfobacterota, whereas in the feces, the dominant phyla were Proteobacteria and Bacteroidetes. These findings were consistent with those previously observed in the cecum of broiler chickens. The relative abundance of several bacterial species increased following DON administration, including Lombutia, Collodextriobacter, and Desulfovibrio, within the rat cecal flora; however, a corresponding decrease was observed in Parasutherella, Pascalectobacterium, and Bacteroides within the fecal flora.

Following exposure to contaminated diets, a notable reduction in the prevalence of Firmicutes was observed in the ileum. A reduction in the abundance of Desulfobacterota improved intestinal bacterial imbalance, suppressed intestinal inflammation, and enhanced intestinal barrier function [52]. This was confirmed by the elevated expression of ZO-1 and occludin, which is consistent with the results observed in this study. In contrast, Desulfobacterota were exclusively identified in the healthy group [53].

Bacilli, Bacteroidetes, Proteobacteria, and Actinomycetota were the most prevalent phyla detected within the colonic compartments. Bisphenol exposure reduced community richness across all three compartments, except for two higher doses in the descending colon, which showed an increase in richness [54]. These findings support our observations that Proteobacteria and Bacteroidetes were the predominant phyla in the fecal samples. At the genus level, the dominant bacterial genera in the cecum were Romboutsia, Colidextribacter, and Desulfovibrio, whereas Parasutterella, Phascolarctobacterium, and Bacteroides were increased in fecal samples. Accordingly, the Bacteroides genus is a beneficial microbe in the context of the gut microbiota. The genus Romboutsia has been identified as a health indicator in humans, with a significant decline in this genus observed in the mucosa, associated with the formation of intestinal polyps [55,56]. Furthermore, DON increases levels of harmful microbes, including Roseburia, Bifidobacterium, and Turicibacter, within the intestine. These shifts in microbial composition may contribute to weight loss in rats. Phascolarctobacteria can produce short-chain fatty acids, including acetate and propionate, which may influence the metabolic state and mood of the host [57]. Phascolarctobacterium (1.6%) is toxic [13]. The genus Romboutsia was identified as a result of probiotic-induced alterations in the gut microbiota, which reduced the severity of pancreatitis and associated sepsis in an experimental rat model of acute pancreatitis. In the conventional anti-mildew treatment group, a notable presence of Desulfovibrio was observed in the jejunum and ileum. Desulfovibrio is a member of the sulfate-reducing bacteria (SRB) family that can stimulate the production of lipopolysaccharides (LPS) and is linked to the inflammatory properties of the microbiota [58]. Therefore, altering the gut structure through the control of Desulfovibrio in the small intestine may effectively reduce inflammatory responses. The genus Parasutterella is a fundamental component of the gut microbiota in humans and mice. Furthermore, there is an established correlation between the presence of Parasutterella and positive health outcomes in ecology [59]. However, similar to the majority of core microbes present in the gastrointestinal tract, our understanding of the biology of Parasutterella and its role in intestinal ecology is limited. 

The identified metabolites in various tissue samples obtained from the control and toxin-treated rats showed distinct spectral phenotypes, indicating discrepancies in their metabolic profiles. Heatmap visualization of metabolites with significant differences in all mycotoxin-treated groups showed a predominant increase in liver, kidney, and ileum metabolites, and a decrease in blood and fecal metabolites, in agreement with the results of the orthogonal projections to the latent structure discriminant analysis. Mycotoxin metabolomics may effectively elucidate the interrelationship between a typical diet and a diet contaminated with these toxins. Toxin studies in rat models should be conducted in controlled environments with reduced environmental variation and consistent sample handling. In the present study, a comprehensive experimental design was employed, wherein the potential influences of sex, age, and breed were rigorously controlled, and an array of parameters, including growth performance and biochemical, histological, and microbiome profiles in different tissues, were observed. Phenylalanine and tryptophan, which are commonly expressed in various tissues, are important for amino acid biosynthesis, making them vital dietary components for the structural integrity and functionality of numerous proteins and enzymes. Phenylalanine is primarily metabolized to tyrosine, which is subsequently transformed into other compounds, including dopamine, serotonin, and epinephrine, which are implicated in diverse biological processes, such as exercise, mood regulation, food intake regulation, and melanogenesis. Furthermore, tryptophan plays several roles in the body, including protein, serotonin, and kynurenine synthesis. It has been demonstrated that the tryptophan availability in the brain can be enhanced by carbohydrate ingestion and reduced by protein ingestion. These findings suggest that the availability of tryptophan, a mycotoxin feeder metabolite, is somewhat altered by dietary intake. Phenylalanine, tyrosine, and tryptophan biosynthesis was identified as significantly enriched KEGG pathways in a transcriptomic analysis of the intestinal effects of different probiotic strains that showed efficacy against DON-induced tissue changes [60]. In the present study, we also reported that ether lipid metabolism, glycerophospholipid metabolism, and phenylalanine metabolism affects the pathways targeted as potential biomarkers of DON in rats. Phenylalanine, tyrosine, and tryptophan biosyntheses were the most prominent pathways identified in this study.

## 5. Conclusions

In conclusion, we demonstrated the effects of DON on animal growth, damage to multiple organs, and disruption of metabolites and gut flora. Among these metabolites, phenylalanine and tryptophan are potential biomarkers in rats. The observed alterations in the gut microbiota following DON exposure suggest its potential involvement in intestinal dysfunction and growth inhibition. The disparate trends observed in the bacterial communities of the cecum and feces may serve as indicators of the degree of DON-induced toxicity. The gut microbiota, particularly *Romboutsia*, *Colidextribacter*, and *Desulfovibrio* in the cecum, and *Parasutterella*, *Phascolarctobacterium*, and *Bacteroides* in the feces, showed changes as a result of DON toxicity in rats. Beneficial bacteria and food sources can help to improve the healthy balance of the gut microbiota, which can lead to an improved response of the host to toxins. However, further experiments are needed to support the results of this study.

## Figures and Tables

**Table 1:** Effects of increasing DON ingestion for 4 weeks on blood biochemistry in rats ^1^.

Parameters ^2^	Control	T1	T2	*p* Value
GLU, mg/dL	211 ± 24.1	166 ± 27.0	185 ± 17.8	0.4189
CREA, mg/dL	0.28 ± 0.07 ^a^	0.10 ± 0.00 ^b^	0.18 ± 0.02 ^ab^	0.0413
BUN, mg/dL	17.6 ± 0.60	17.8 ± 1.77	19.40 ± 1.69	0.6425
BUN/CREA	83.2 ± 21.43	178 ± 17.72	122 ± 32.35	0.0545
PHOS, mg/dL	13.06 ± 0.94	11.64 ± 0.73	11.22 ± 0.57	0.2399
CA, mg/dL	11.46 ± 0.24	10.90 ± 0.33	11.24 ± 0.64	0.6685
TP, g/dL	6.66 ± 0.05	7.22 ± 0.37	7.04 ± 0.37	0.4387
ALB, g/dL	3.96 ± 0.12	4.42 ± 0.33	4.10 ± 0.24	0.4205
GLOB, g/dL	2.70 ± 0.10	2.80 ± 0.18	2.94 ± 0.14	0.5122
ALB/GLOB	1.48 ± 0.10	1.60 ± 0.18	1.40 ± 0.04	0.5229
ALT, U/L	88.00 ± 7.40	104.20 ± 15.57	105.8 ± 24.63	0.7309
ALKP, U/L	253.60 ± 21.94 ^a^	166.60 ± 13.43 ^b^	180.80 ± 35.39 ^b^	0.0172
GGT, U/L	2.00 ± 2.00	0.00 ± 0.00	0.00 ± 0.00	0.3966
TBIL, mg/dL	0.44 ± 0.11	1.18 ± 0.50	1.06 ± 0.79	0.5994
CHOL, mg/dL	72.20 ± 1.62	67.00 ± 2.05	64.80 ± 8.99	0.6217
AMYL, U/L	1787.8 ± 80.27	1686.4 ± 53.20	1889.4 ± 122.82	0.3163
LIPA, U/L	172.4 ± 17.79	173.0 ± 11.26	184.4 ± 12.51	0.7990

^1^ Control, basal diet; T1, basal diet + 0.02 mg/L DON; T2, basal diet + 0.2 mg/L DON. ^2^ GLU, glucose; CREA, creatinine; BUN, blood urea nitrogen; PHOS, phosphate; CA, calcium; TP, total protein; ALB, albumin; GLOB, globulin; ALT, alanine aminotransferase; ALKP, alkaline phosphate; GGT, gamma-glutamyl transferase; TBIL, total bilirubin; CHOL, cholesterol; AMYL, amylase; LIPA, lipase. ^a,b^ Different superscript letters indicate that the variables within a row differed significantly (*p* < 0.05).

## Data Availability

Dataset available on request from the authors.
